# Exposure-Based, ‘Like-for-Like’ Assessment of Road Safety by Travel Mode Using Routine Health Data

**DOI:** 10.1371/journal.pone.0050606

**Published:** 2012-12-05

**Authors:** Jennifer S. Mindell, Deborah Leslie, Malcolm Wardlaw

**Affiliations:** 1 Research Department of Epidemiology and Public Health, UCL (University College London), London, United Kingdom; 2 Centre for Physical Activity and Nutrition Research, Deakin University, Burwood, Australia; 3 Edinburgh, United Kingdom; Tehran University of Medical Sciences, Iran (Republic of Islamic)

## Abstract

**Background:**

Official reports on modal risk have not chosen appropriate numerators and denominators to enable like-for-like comparisons. We report age- and sex-specific deaths and injury rates from equivalent incidents in England by travel mode, distance travelled and time spent travelling.

**Methods:**

Hospital admissions and deaths in England 2007–2009 were obtained for relevant ICD-10 external codes for pedestrians, cyclists, and car/van drivers, by age-group and sex. Distance travelled by age-group, sex and mode in England (National Travel Survey 2007–2009 data) was converted to time spent travelling using mean trip speeds. Fatality rates were compared with age-specific Netherlands data.

**Results:**

All-age fatalities per million hours’ use (f/mhu) varied over the same factor-of-three range for both sexes (0.15–0.45 f/mhu by mode for men, 0.09–0.31 f/mhu for women). Risks were similar for men aged 21–49 y for all three modes and for female pedestrians and drivers aged 21–69 y. Most at risk were: males 17–20 y (1.3 f/mhu (95% CI 1.2–1.4)) for driving; males 70+ (2.2 f/mhu(1.6–3.0)) for cycling; and females 70+ (0.95 f/mhu (0.86–1.1)) for pedestrians. In general, fatality rates were substantially higher among males than females. Risks per hour for male drivers <30 y were similar or higher than for male cyclists; for males aged 17–20 y, the risk was higher for drivers (33/Bn km (30–36), 1.3 f/mhu (1.2–1.4)) than cyclists (20/Bn km (10–37), 0.24 f/mhu (0.12–0.45)) whether using distance or time. Similar age patterns occurred for cyclists and drivers in the Netherlands. Age-sex patterns for injuries resulting in hospital admission were similar for cyclists and pedestrians but lower for drivers.

**Conclusions:**

When all relevant ICD-10 codes are used, fatalities by time spent travelling vary within similar ranges for walking, cycling and driving. Risks for drivers were highest in youth and fell with age, while for pedestrians and cyclists, risks increased with age. For the young, especially males, cycling is safer than driving.

## Introduction

Travel can provide many health benefits through access to facilities, goods, and people. Active travel, i.e. walking and cycling, can make additional large contributions to population health: daily repetitive and necessary physical activity, such as commuting, can have the greatest health benefits as these are more successful and durable over long periods [1]. Cycling as a mode of commuting has additional advantages for society, including reducing carbon emissions, noise levels, and congestion on roads and other public transport systems [2], [3]. Increasing active travel in England and Wales is estimated to save £17 bn in healthcare costs alone [4]. Despite these documented benefits and some increases in cycling in several cities with specific interventions [5], [6], the UK has no nationwide cycling revival.

Perceived road danger is a strong disincentive to cycling [7]; many cyclists do not ride on the road due to safety concerns [8]. However, research regarding the safety of cycling tends to be distorted by a number of substantial errors which are found repeatedly in published papers and policy documents. These fall into three main categories:

- not accounting for different types of journey undertaken in each mode, notably long-distance car travel, which has no comparison in walking or cycling, unless train travel is included;
- choice of a misleading denominator, such as comparing cycling fatality rates internationally using population size as the denominator [9], [10];
- not selecting comparable numerators, that is, failing to include all transport casualties and exclude non-transport casualties.

Concerning the first two errors, the importance of using the most appropriate measure of exposure has been demonstrated in inter-country comparisons [10], [11]. Risk by distance travelled does not capture large differences in average speed, which enable differential mobility for drivers, cyclists, and pedestrians. As the speed differential between cars and bicycles is not great for local journeys, time-based comparison minimises the distorting impact of misleading comparisons with long distance car journeys. The average “main driver” travels 7,034 miles, plus a further 1,254 miles as a passenger; the average cyclist rides about 830 miles annually [12].

Surveys from many countries show that time spent travelling has remained fairly constant over time at about one hour per day, despite large changes in modal split [13]. This supports the use of risk based on time as being most appropriate for comparing modes with different average speeds.

**Figure 1:**
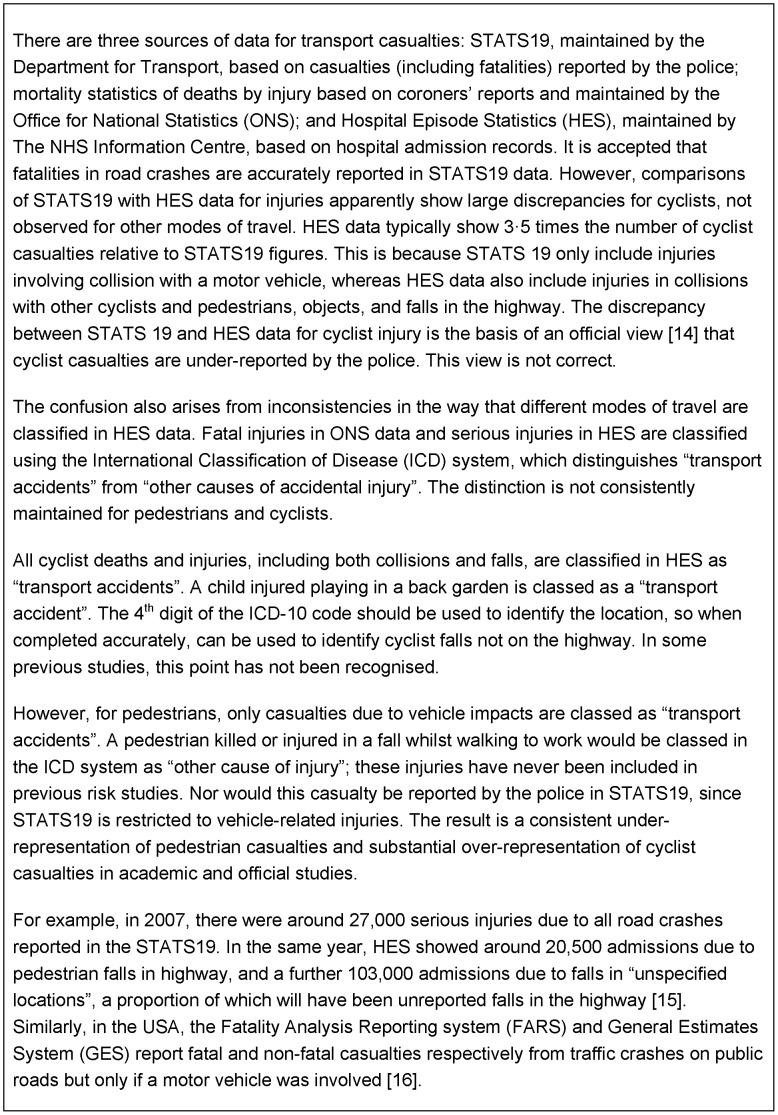
Sources of data on transport injuries and fatalities.

The third category of error is to include off-highway falls and children injured at play as cycling transport injuries, whilst omitting all falls of pedestrians (whether on- or off-highway). This substantially overstates cycling injuries and understates pedestrian injuries (Figure 1).

In the UK, most drivers are middle-aged adults, with almost half being female, while cycling is male-dominated and has traditionally been considered predominantly an activity of youth: 24% of serious casualties among cyclists are <20 y old, compared with 12% of serious casualties among drivers [9]. The increased risk-taking associated with both young age and male sex are well-known and confound comparisons of cycling and driving.

The two sources of data on transport injuries, STATS 19 and Hospital Episode Statistics (HES), include different categories of incident but neither is totally comparable across different travel modes (Figure 1). Other difficulties for like-with-like comparisons are described in the methods section below. Not all these problems are amenable to investigation using routine data, but some can be addressed.

Because of these problems, we undertook a study based on mortality data and HES, taking account of the inconsistencies in the classification of the data with the aim of filling this important gap. This paper provides age- and sex-specific deaths and injuries in England by travel mode, using both distance and time as measures of exposure. We considered both the ICD external codes included in official travel risk data and those currently excluded but required for comparative assessment, successfully providing more accurate estimates on which to base comparison of travel risks by mode. We also compared our findings with data from the Netherlands.

## Methods

### Definition of “risk”

In this paper, “risk” is defined as the observed number of casualties per unit of exposure. We considered two severities of injury - fatal and hospital admission - and two units of exposure: distance travelled (billion km) and time spent travelling (million hours). Most emphasis was given to risk as “fatalities per million hours’ use” (f/mhu). “Hospital admission” does not capture differing severities of injury between modes of travel: mean length of hospital stay after traffic collision was 2.9 d for cyclists and drivers, but 4.7 d for pedestrians [17].

### Denominator Data

The National Travel Survey (NTS), the primary source of data on personal travel patterns in Great Britain, includes 15,000 households each year. The nationally-representative sample is selected annually using a stratified two-stage random probability sample of private households. Data collection comprises a face-to-face interview and a one-week self-completed written travel diary [12]. The Department for Transport also measures vehicle traffic using on-road manual and automatic traffic counts. During the last decade, the bicycle traffic estimates from these two independent sources have agreed within 10% [18]. However, both data sources exclude travel on routes not usable by motor vehicles, thus underestimating travel by foot and by bicycle.

The Department for Transport NTS team provided the authors with aggregated per capita distance travelled by age-group, sex and mode in England from NTS data for each year 2007–2009, weighted to be nationally-representative. Total distance travelled by mode over the three years for each age- and sex-specific group was obtained by multiplying the distance for each age/sex group by the estimated population for that group and year.

Data for mean trip speeds (walking 4·2 km/h, cycling 12·2 km/h, driving 39 km/h) were available from the DfT [19]. More detailed data were not available, so the same speeds were used for all age- and sex-specific groups.

### Numerator Data

#### Defining ‘travel injuries/fatalities’

ICD-10 external codes were grouped by type of incident; the fourth digit was used to distinguish between different locations (e.g. on-highway, off-highway, or unspecified) or other details. Table S1 shows the full specification of the ICD-10 codes included in each group, and identifies which groups are included/excluded from STATS19 statistics and NHS transport-related casualty data. Data were sought for pedestrians, cyclists, and car/van drivers but not vehicle passengers, using the age-groups for which NTS travel data were provided. Non-transport casualties, such as pedestrian falls at work or off-highway cyclist falls, were excluded as much as possible by specifying the four-digit ICD-10 codes required (see table S1). This enabled compatibility with denominator data for distance travelled on highways only.

#### Casualty data

Numbers of hospital admissions, from Hospital Episodes Statistics (HES) data [17], and deaths in England aggregated over 2007–2009 were provided by the London Health Observatory for each group of ICD codes specified in Table S1, by age-group and sex. The three year period was used to improve precision and data non-disclosiveness, especially for cycling.

### Netherlands Data

Published age-specific fatality rates by distance travelled for car occupants and cyclists from the Netherlands [20] were converted to f/mhu using mean travel speeds produced by the Centraal Bureau voor de Statistiek [21].These were: walking 5.5 km/h; cycling 13.4 km/h; and driving 44.4 km/h.

### Analysis

Age- and sex-specific fatality and injury rates for England were calculated by dividing the casualty data (numbers aggregated over the three years) for each age- and sex-specific group by the distance travelled by that group over the three-year period. This was also calculated for all persons by age-group. These rates per unit distance were then converted into rates per unit time using mean trip speed data for each mode, for both countries.

All analyses were conducted in Excel 2010. The 95% confidence intervals for Poisson parameters were calculated for the English data using the formulae for weighted sums [22].

### Data Sharing Statement

The aggregated age- and sex-specific data can be obtained from the data holders or, with the data holders’ permission, from the authors. Anonymised National Travel Survey data are available from the UK Data Service. Mortality data and Hospital Episodes Statistics data are available from the Health and Social Care Information Centre.

## Results

### England

The ICD system allows for incomplete data to be coded in more general categories such as “unspecified occupant in unspecified transport accident” (eg V49.9). These are known as “dustbin” codes. All results were affected by dustbin coding. For drivers, an unknown number of passengers will have been included as “unspecified occupants”. For cyclists, an unknown number of non-transport casualties will have been included in code V19.8. The results for drivers and cyclists should thus be seen as pessimistic. For pedestrians, an unknown number of on-highway falls will have been excluded as “falls in unspecified location” (eg W19.9). The results for walking are thus underestimates.

Caution is therefore advised when considering results for falls in highway for walking and cycling, especially in older age groups. The results apparently show more cyclist deaths due to falls than traffic crashes in age groups 50–59 y and 70+y (Tables 1 and 2) but it is not certain that all these casualties were transport-related. In contrast, for walking, some of the 434 fatal falls in unspecified location for the 70+y group will have been transport-related.

**Table 1:** Fatality numbers and rates per distance travelled, by travel mode, age, and type of incident, Males, England 2007–2009.

Mode		Summary description	Age-group								
			<17	17–20	21–29	30–39	40–49	50–59	60–69	70+	ALL
**Drive**	3 yr distance (Mn km)		14	11,354	59,462	100,915	128,207	102,998	67,160	34,383	504,493
	Driver Collision Fatality	Drive-RTA	*3*	114	164	108	91	71	66	122	739
	Driver Single vehicle fatality	Drive-RTA (single vehicle)	*6*	158	221	122	76	51	28	35	697
	Unspecified occupant unspecifiedaccident^a^	Drive-RTA (unspecified)	*36*	101	122	65	51	47	24	48	494
	**On-highway fatality rate (per Bn km)^bc^**		**3,214** d	**33**	**8.5**	**2.9**	**1.7**	**1.6**	**1.8**	**6.0**	**3.8**
	*95% CIs*		*2,345*–*4,301*	*30*–*36*	*7.8*–*9.3*	*2.6*–*3.3*	*1.5*–.*9*	*1.4*–*1.9*	*1.5*–*2.1*	*5.2*–*6.8*	*3.7*–*4.0*
**Cycle**	3 yr distance (Mn km)		925	497	1,151	1,555	2,115	1,025	520	258	8,046
	3 yr Cycle-RTA (n)	Collision	36	6	20	33	26	23	20	21	185
	3 yr Cycle-fall (n)	On-highway^e^	6	4	9	17	10	27	10	26	109
		*Off-highway* f	*2*	*0*	*1*	*1*	*1*	*0*	*3*	*0*	*8*
	**On-highway fatality rate (per Bn km)** e		**45**	**20**	**25**	**32**	**17**	**49**	**58**	**182**	**37**
	*95% CIs*		*33*–*61*	*10*–*37*	*17*–*36*	*24*–*42*	*12*–*24*	*36*–*64*	*39*–*82*	*134*–*243*	*32*–*41*
**Walk**	3 yr distance (Mn km)		5,132	1,464	2,951	2,827	2,638	2,342	2,115	1,561	21,030
	3 yr Walk-RTA (n)	Specified	70	50	75	73	62	54	60	185	629
		Unspecified	31	21	44	39	47	47	30	96	355
	3 yrn Walk-fall (n)	On Highway	0	2	4	16	36	44	54	148	304
		*Other specified location* f	*4*	*3*	*11*	*9*	*13*	*16*	*17*	*45*	*118*
		*Unspecified Location^fg^*	*2*	*1*	*5*	*4*	*11*	*32*	*42*	*434*	*531*
	**On-highway fatality rate (per Bn km)** g		**20**	**50**	**42**	**45**	**55**	**62**	**68**	**275**	**61**
	*95% CIs*		*16*–*24*	*39*–*63*	*35*–*50*	*38*–*54*	*46*–*65*	*52*–*73*	*57*–*80*	*249*–*302*	*58*–*65*

a This group may also contain passengers but probably notmany as “unspecified occupant” was rare for collisions.These have been included in the fatality rate estimate.

b Estimates are likely to be a little too high due to the assumption that unspecified occupants are all drivers: an unknown proportion would have been passengers, especially for females.

c Note that these averages include both local roads and motorways/multi-lane divided roadways, where fatality rates are an order of magnitude lower than general purpose roads, but data are not available by age and sex.

d This figure is greatly exaggerated by under-measurement of under-aged driving.

e These figures are too high as V19.8 is a dustbin code, including some off-highway falls.

f Not included in the fatality rate.

g Estimates are too low, as some ‘unspecified location’ deaths will have been on-highway.

**Table 2:** Fatality numbers and rates per distance travelled, by travel mode, age, and type of incident, Females, England 2007–2009.

Mode			Summary description	Age-group								
				<17	17–20	21–29	30–39	40–49	50–59	60–69	70+	ALL
**Drive**	3 yr distance (Mn km)			8	9,816	44,170	61,447	74,397	48,749	24,998	11,466	275,051
	Driver CollisionFatality		Drive-RTA	0	28	57	20	37	21	21	57	241
	Driver Singlevehicle fatality		Drive-RTA (single vehicle)	0	30	36	20	16	16	11	17	146
	Unspecifiedoccupantunspecifiedaccident^a^		Drive-RTA (unspecified)	27	47	35	24	29	25	25	40	252
	**On-highway fatality rate (per Bn km)^bc^**			**3,375** d	**11**	**2.9**	**1.0**	**1.1**	**1.3**	**2.3**	**9.9**	**2.3**
	*95% CIs*			*2.224*–*4,910*	*9*–*13*	*2.4*–*3.4*	*0.8*–*1.3*	*0.9*–*1.4*	*1.0*–*1.6*	*1.7*–*3.0*	*8.2*–*12*	*2.1*–*2.5*
**Cycle**	3 yr distance (Mn km)			286	74^e^	391	399	440	288	118	60	2,056
	3 yr Cycle-RTA		Collision	5	5	5	7	8	4	2	3	39
	3 yr Cycle-fall		On-highway^f^	0	0	1	3	2	4	2	0	12
			*Off-highway* g	*0*	*0*	*0*	*0*	*0*	*0*	*0*	*0*	*0*
	**On-highway fatality rate (per Bn km)** f			**18**	**67**	**15**	**25**	**23**	**28**	**34**	**50**	**25**
	*95% CIs*			*6*–*41*	*22*–*157*	*6*–*33*	*12*–*46*	*11*–*42*	*12*–*55*	*9*–*87*	*10*–*146*	*18*–*33*
**Walk**	3 yr distance (Mn km)			5,022	1,300	3,184	3,568	3,425	2,483	2,024	1,674	22,680
	3 yr Walk-RTA (n)	Specified		47	15	15	19	29	24	31	159	339
		Unspecified		19	6	15	12	19	11	11	103	196
	3 yrn Walk-fall (n)	On Highway		0	0	1	4	8	5	7	117	142
		*Other specified location* g		*0*	*0*	*1*	*1*	*1*	*6*	*2*	38	49
		*Unspecified Location^gh^*		*0*	*0*	*0*	*1*	*5*	*9*	*27*	634	676
	**On-highway fatality rate (per Bn km)** g			^13^	**16**	**10**	**10**	**16**	**16**	**24**	**226**	**30**
	*95% CIs*			*10*–*17*	*10*–*25*	*7*–*14*	*7*–*14*	*12*–*21*	*12*–*22*	*18*–*32*	*204*–*250*	*28*–*32*

a This group may also contain passengers but probably notmany as “unspecified occupant” was rare for collisions.These have been included in the fatality rate estimate.

b Estimates are likely to be a little too high due to the assumption that unspecified occupants are all drivers: an unknown proportion would have been passengers, especially for females.

c Note that these averages include both local roads and motorways/multi-lane divided roadways, where fatality rates are an order of magnitude lower than general purpose roads, but data are not available by age and sex.

d This figure is greatly exaggerated by under-measurement of under-aged driving.

e The base for this was much smaller than distances for other ages, sex, and travel modes.

f These figures are too high as V19.8 is a dustbin code, including some off-highway falls.

g Not included in the fatality rate.

h Estimates are too low, as some ‘unspecified location’ deaths will have been on-highway.

For both walking and cycling by under-17 s, only a very small number of deaths due to falls occurred. It is thus difficult to draw conclusions about fatal risk without a longer sample period. For serious injuries, i.e. those resulting in hospital admission, the risk of falls appears to be higher for cycling than walking (Tables 3 and 4). While this may be the case, the large proportion of “unspecified location” walking falls must be noted. In addition, for child cyclists, distinguishing falls at play in the street from falls during travel is particularly difficult. The risks for under-age drivers are extremely high. Although unlicensed drivers face high risks - and impose high risks on others [23] - the results in this study are over-estimates due to under-recording of the denominator resulting from the difficulty of measuring an illegal activity. These results must be treated with caution.

**Table 3:** Hospital admission numbers and rates per distance travelled, by travel mode, age, and type of incident, Males, England 2007–2009.

Mode		Summary description	Age-group								All ages
			<17	17–20	21–29	30–39	40–49	50–59	60–69	70+	
**Drive**	3 yr distance (Mn km)		14	11,354	59,462	100,915	128,207	102,998	67,160	34,383	470,110
	Driver Collision	Drive-RTA	61	1,202	2,127	1,745	1,706	1,284	964	1,182	10,271
	Driver Single vehicle	Drive-RTA (single vehicle)	76	1,447	2,053	1,216	888	522	358	634	7,194
	Unspecified occupant unspecified accident^a^	Drive-RTA (unspecified)	269	538	745	474	463	304	221	342	3,356
	**Hospital admission rate (per Bn km)^bc^**		**29,000** d	**281**	**82.8**	**34.0**	**23.8**	**20.5**	**23.0**	**62.8**	**41.3**
	*95% CIs*		*26,247*–*31,963*	*271*–*291*	*81*–*85*	*33*–*35*	*23*–*25*	*20*–*21*	*22*–*24*	*60*–*65*	*44*–*45*
**Cycle**	3 yr distance (Mn km)		925	497	1,151	1,555	2,115	1,025	520	258	8,046
	3 yr Cycle-RTA (n)	Collision	1,752	429	838	1,031	1,128	621	324	241	6,364
	3 yr Cycle-fall (n)	On-highway^e^	5,212	883	1,357	1,729	1,589	1,014	636	484	12,904
	**Hospital admission rate (per Bn km)** e		**7,529**	**2,642**	**1,906**	**1,775**	**1,285**	**1,595**	**1,845**	**2,815**	**2,395**
	*95% CIs*		*7,345*–*7,708*	*2,501*–*2,789*	*1,828*–*1,988*	*1,709*–*1,842*	*1,237*–*1,334*	*1,519*–*1,674*	*1,730*–*1,966*	*2,613*–*3,027*	*2,361*–*2,429*
**Walk**	3 yr distance (Mn km)		5,132	1,464	2,951	2,827	2,638	2,342	2,115	1,561	21,030
	3 yr Walk-RTA (n)	Specified	4,692	1,283	2,001	1,595	1,477	1,095	883	1,550	14,576
		Unspecified	411	128	223	177	216	167	104	224	1,650
	3 yrn Walk-Fall (n)	On-Highway	1,940	1,215	2,656	3,022	4,450	4,445	4,917	12,607	35,252
		*Unspecified Location^fg^*	*16,923*	*4,571*	*10,173*	*10,425*	*13,046*	*12,824*	*14,311*	*58,613*	*140,886*
	**Hospital admission rate (per Bn km)** g		**1,372**	**1,794**	**1,654**	**1,696**	**2,329**	**2,437**	**2,791**	**9,213**	**2,448**
	*95% CIs*		*1,341*–*1,405*	*1,726*–*1,864*	*1,608*–*1,701*	*1,648*–*1,744*	*2,271*–*2,388*	*2,374*–*2,501*	*2,721*–*2,864*	*9,063*–*9,365*	*2,427*–*2,469*

a This group may also contain passengers but probably notmany as “unspecified occupant” was rare for collisions.These have been included in the fatality rate estimate.

b Estimates are likely to be a little too high due to the assumption that unspecified occupants are all drivers: an unknown proportion would have been passengers.

c Note that these averages include both local roads and motorways/multi-lane divided roadways, where fatality rates are an order of magnitude lower than general purpose roads, but data are not available by age and sex.

d This figure is greatly exaggerated by under-measurement of under-aged driving.

e These figures are too high as V19.8 is a dustbin code, including some off-highway falls.

f Not included in the fatality rate.

g Estimates are too low, as some ‘unspecified location’ deaths will have been on-highway.

**Table 4:** Hospital admission numbers and rates per distance travelled, by travel mode, age, and type of incident, Females, England 2007–2009.

Mode			Summary description			Age-group								All ages
						<17	17–20	21–29	30–39	40–49	50–59	60–69	70+	
**Drive**	3 yr distance (Mn km)					8	9,816	44,170	61,447	74,397	48,749	24,998	11,466	275,051
	Driver Collision				Drive-RTA	65	686	1,510	1,223	1,152	898	574	801	6,909
	Driver Single vehicle				Drive-RTA (single vehicle)	18	524	732	465	351	285	168	381	2,924
	Unspecified occupantunspecified accident^a^				Drive-RTA (unspecified)	270	337	629	420	243	229	197	485	2,810
	**Hospital admission rate (per Bn km)^bc^**					**44,125** d	**158**	**65**	**34**	**24**	**29**	**37.6**	**145**	**46**
	*95% CIs*					*39,641*–*48,977*	*150*–*166*	*63*–*67*	*33*–*36*	*22*–*25*	*27*–*31*	*35*–*40*	*138*–*153*	*45*–*47*
**Cycle**	3 yr distance (Mn km)					286	74^e^	391	399	440	288	118	60	2,056
	3 yr Cycle-RTA (n)				Collision	296	85	247	225	209	161	93	75	1,391
	3 yr Cycle-fall (n)				On-highway^f^	1,259	66	314	354	374	430	304	189	3,290
	**Hospital admission rate (per Bn km)** f					**5,445**	**2,035**	**1,435**	**1,451**	**1,325**	**2,050**	**3,358**	**4,405**	**2,277**
	*95% CIs*					*5,178*–*5,723*	*1,724*–*2,387*	*1,319*–*1,559*	*1,335*–*1,574*	*1,220*–*1,437*	*1,888*–*2,223*	*3,036*–*3,706*	*3,890*–*4,970*	*2,212*–*2,343*
**Walk**	3 yr distance (Mn km)					5,022	1,300	3,184	3,568	3,425	2,483	2,024	1,674	22,680
	3 yr Walk-RTA (n)			Specified		2,658	576	931	702	688	698	663	2,096	9,012
				Unspecified		248	43	65	54	68	55	65	253	851
	3 yrn Walk-fall (n)			On-highway		1,126	557	1,355	1,639	2,570	4,161	6,120	23,307	40,835
				*Unspecified* *Location* ^gh^		*9,425*	*3,133*	*8,540*	*8,588*	*9,397*	*13,462*	*18,803*	*126,098*	*197,446*
	**Hospital admission rate (per Bn km)** h					**803**	**905**	**738**	**671**	**971**	**1,979**	**3,383**	**15,326**	**2,235**
	*95% CIs*					*778*–*828*	*854*–*958*	*709*–*769*	*645*–*699*	*938*–*1,005*	*1,924*–*2,035*	*3,304*–*3,465*	*15,139*–*15,515*	*2,216*–*2,255*

a This group may also contain passengers but probably notmany as “unspecified occupant” was rare for collisions.These have been included in the fatality rate estimate.

b Estimates are likely to be a little too high due to the assumption that unspecified occupants are all drivers: an unknown proportion would have been passengers.

c Note that these averages include both local roads and motorways/multi-lane divided roadways, where fatality rates are an order of magnitude lower than general purpose roads, but data are not available by age and sex.

d This figure is greatly exaggerated by under-measurement of under-aged driving.

e The base for this was much smaller than distances for other ages, sex, and travel modes.

f These figures are too high as V19.8 is a dustbin code, including some off-highway falls.

g Not included in the fatality rate.

h Estimates are too low, as some ‘unspecified location’ deaths will have been on-highway.

**Figure 2:**
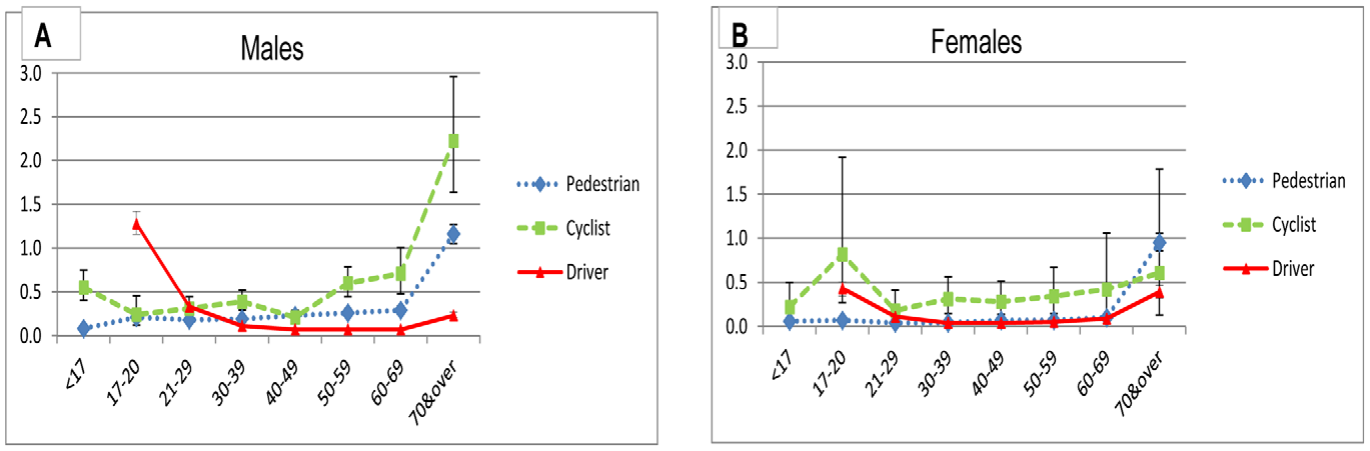
Fatality rates per million hours’ use by travel mode, age, and sex. a. Males. b. Females. f/mhu: fatality rate per million hours use in England, 2007–2009.

The principal outcome, fatalities per million hours’ use, varied substantially by age and sex. For most age groups, the rate was within a similar range for all three modes. Disaggregated by age, risks for all three modes were similar for men aged 21–49 and for female pedestrians and drivers aged 21–69 y (Figure 2). The change in risk with age followed different patterns by mode. For drivers, the risk was highest in youth, falling by a factor of 20 into middle age before rising again for 70+. For walking and cycling, the variation in risk with age was much less, generally increasing with age. The exception was female cyclists aged 17–20 y, although this was a small group and the result could be chance fluctuation, given the small casualty numbers and distances cycled. The group most at risk for each mode were: male drivers aged 17–20 y (1.3 f/mhu (95% CI 1.2–1.4)); male cyclists aged 70+ (2.2 f/mhu (1.6–3.0)): and female pedestrians aged 70+ (0.95 f/mhu (0.86–1.1), Figure 2). In general, fatality rates were substantially higher amongst males than females, except for drivers aged 60+. Risks per hour for male drivers <30 y were similar or higher than for male cyclists; for those aged 17–20 y, the risk was higher for drivers (33/Bn km (95% CI 30–36), 1·3 f/mhu (1.2–1.4)) than cyclists (20/Bn km (10–37), 0.55 f/mhu (0.4–0.75)) using distance or time.

Overall rates of hospital admissions per mhu were similar in men and women, but varied more widely by mode than fatality rates did (Table 5). A similar U-shaped pattern with age was seen in both sexes and for all three modes. Males were at higher risk than females as drivers aged 17–29 y, as cyclists aged 17–39 y, and as pedestrians aged 17–59 y.

**Table 5:** Hospital admission rates per million hours travel (mhu^a^) by travel mode, age, and sex, England 2007–2009.

Mode	Age-group								All ages
	<17	17–20	21–29	30–39	40–49	50–59	60–69	70+	
**Men**									
Drive^b^	1,131^c^	11	3.2	1.3	0.9	0.8	0.9	2.5	1.6
*95% CIs*	*1,024*–*1,247*	*11*–*11*	*3.1*–.*3.3*	*1.3*–*1.4*	*0.9*–*1.0*	*0.8*–*0.8*	*0.9*–*0.9*	*2.3*–*2.6*	*1.6*–*1.6*
Cycle^d^	92	32	23	22	16	20	23	34	29
*95% CIs*	*90–94*	*31–34*	*22–24*	*21–22*	*15–16*	*19–20*	*21–24*	*32–37*	*29–30*
Walk^e^	5.8	7.5	6.9	7.1	9.8	10	12	39	10
*95% CIs*	*5.6–5.9*	*7.2–7.8*	*6.8–7.1*	*6.9–7.3*	*9.5–10*	*10–11*	*11–12*	*38–39*	*10–10*
**Women**									
Drive^b^	1,720^c^	6.2	2.5	1.3	0.9	1.1	1.5	5.7	1.8
*95% CIs*	*1,546–1,910*	*5.8–6.5*	*2.4–2.6*	*1.3–1.4*	*0.9–1.0*	*1.1–1.2*	*1.4–1.6*	*5.4–5.9*	*1.8–1.8*
Cycle^d^	67	25	18	18	16	25	41	54	28
*95% CIs*	*63–70*	*21–29*	*16–19*	*16–19*	*15–18*	*23–27*	*37–45*	*47–61*	*27–29*
Walk^e^	3.4	3.8	3.1	2.8	4.1	8.3	14	64	9.4
*95% CIs*	*3.3–3.5*	*3.6–4.0*	*3.0–3.2*	*2.7–2.9*	*3.9–4.2*	*8.1–8.5*	*14–15*	*64–65*	*9.3–9.5*

a mhu: million hours use, estimated using National Travel Survey average speed for all trips by this mode as not available by age and sex.

b These averages include both local roads and motorways/multi-lane divided roadways, where fatality rates are an order of magnitude lower than general purpose roads, but data are not available by age and sex.

c This figure is greatly exaggerated by under-measurement of under-aged driving.

d These figures are too high as V19.8 is a dustbin code, including some off-highway falls.

e Estimates are too low, as some ‘unspecified location’ deaths will have been on-highway.

### Comparison with the Netherlands

**Figure 3:**
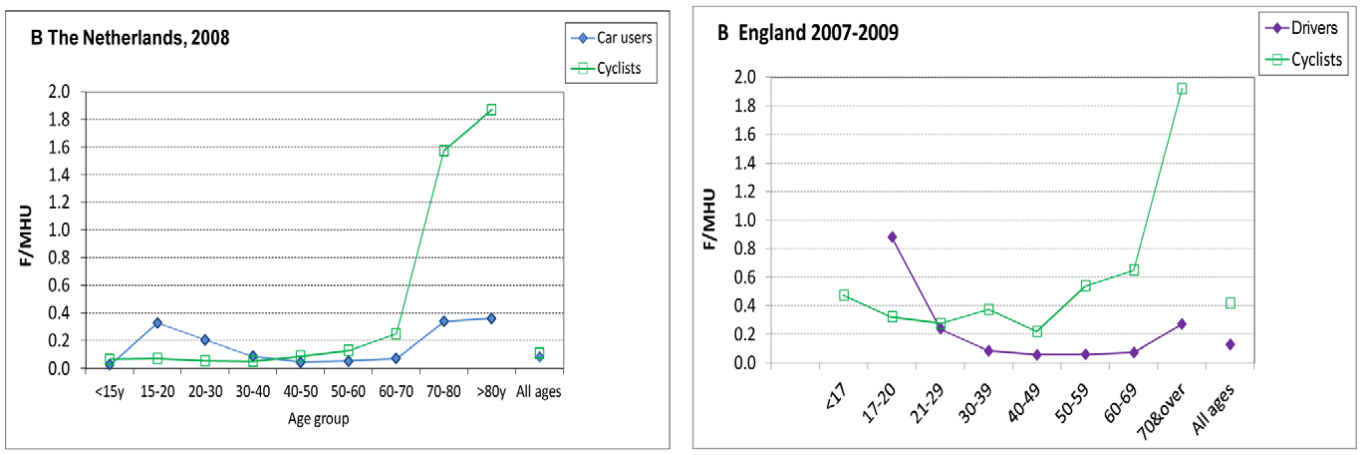
Fatality rates per million hours’ use in the Netherlands and England, by age. a. The Netherlands, 2008. b. England, 2007–2009. There are a number of limitations to these comparisons. First, data for the two countries use slightly different age-groups. Due to inaccuracies in driving data for those below the legal limit for driving, data for the youngest groups are not shown, although they are included in the ‘all ages’ categories. Secondly, fatality rates for the Netherlands are for all car occupants whereas for England they are restricted to drivers (plus small numbers of fatalities for unspecified car occupants), with passengers excluded from both the numerator and denominator. Thus the age-specific rates for the Netherlands underestimate the variability in rate by age of driver. Thirdly, the data shown are the actual ‘all persons’ data from the two countries. Therefore, the English data for cyclists are weighted to the figures for males. English males cycled four times the distance but had six times as many fatalities as women, and spent twice as much time driving as women but had three times as many deaths. For the Netherlands, driving is similarly dominated by males, but cycling distance is equally split between males and females.

Comparing the fatality risk for cyclists and car users/drivers by time between the Netherlands and England (Figure 3), a similar pattern was seen of fatality rates for younger car users/drivers exceeding those for cyclists for people under 30 y. Both countries showed a marked increase in fatalities in those aged 70 y and over, particularly for cyclists. This increase was of the same magnitude in both countries. However, rates in the Netherlands for cyclists <70 y were closer to those for drivers than the equivalent data for England.

## Discussion

### Principal Findings in Relation to Existing Knowledge

Previous assessments showed that the all-ages risks per hour vary by country for all travel modes, with the risk for UK cyclists higher than for drivers or pedestrians [24]. Our study confirms this is so overall. It also reveals the large variations by age and sex, especially for younger drivers and older pedestrians and cyclists. However, the range of risks is similar for each mode. Comparisons with the Netherlands revealed a similar pattern by age in both countries for both cyclists and car users/drivers.

It has not been previously reported that males in England aged 17–20 y face higher risks as drivers than as cyclists, and do not achieve better safety as drivers until their 20 s or 30 s, as has also been shown in the Netherlands [20]. Due to constraints of exposure data being provided by set age-groups, we were unable to determine a precise age at which risk by mode crosses over. However, in view of the risk implied to third parties, it is clearly misleading for official publications to suggest that cycling is relatively hazardous. An American study limited to road traffic crashes involving motor vehicles found that the fatality rate per trip was higher for those aged 15–24 y as vehicle occupants (21.3 deaths per 100 million person-trips) than pedestrians (12.4); although lower than for cyclists (30.9), the 95% CIs for vehicle occupants and cyclists overlapped. It did not distinguish between drivers and passengers, nor between males and females by age-group [16].

The risks calculated in this study are all very low. An individual who cycles 1 hr/d for 40 y at a fatality rate of 34/Bn km would cover about 180,000 km, whilst accumulating only a one in 150 chance of fatal injury. Several studies have shown health benefits at least an order of magnitude greater than fatality risk [25], [26], which cannot be claimed for driving. Our study confirms that pedestrians face higher fatality rates per km travelled (45 fatalities/Bn km in 2007–2009) than cyclists (34/Bn km), yet walking has never been regarded as unduly hazardous. Data from the USA, Germany and the Netherlands have also found higher risks for pedestrians than cyclists per distance travelled, despite the higher risks for non-motor travel in the USA [27]. There is clearly a discrepancy between the popular image of cycling as dangerous and the reality that, in most age groups, it is safe by everyday standards, and yields health benefits that, for the previously sedentary middle-aged, are greater than cessation of cigarette smoking [28].

The situations in other countries are often more favourable to cyclists than shown by this study. Data from the French National Travel Survey and road fatality records reveal that the all-ages average risk per hour of cycling in France(0.3 f/mhu: 150 fatalities, 5.7 Bn km national distance, 13.3 kph, 2008 data) is a little less than in the UK, despite the general absence of cycling infrastructure and a worse safety record for drivers (Personal communication to MW 19/7/2011 from the French Ministry of Ecology, Sustainable Development and Energy).

### Strengths

This is the first study in the UK to provide travel casualty rates by distance travelled and per hour, by mode, by age-group and sex, based on nationally-representative data, to enable unbiased intermodal comparisons for population sub-groups. The main strengths of this study lie in assessing fatal and serious travel injuries from mortality statistics and hospital records respectively rather than police reports; using data by mode, age and sex; and by providing data both including and excluding relevant codes. This shows that official studies based on NHS data exaggerate the risks of cycling by including non-travel injuries (Cycle-fall (off-highway)) and underestimate the risks of walking by excluding pedestrian falls on the highway. These errors combine to sustain or enhance the misleading stereotype that travelling by bicycle is relatively hazardous. A study from Israel found that 63% of bicycle injuries resulting in hospitalization in adults and 73% in children did not involve a motor vehicle [29]. We did not include walking casualties in unspecified locations or off-highway when calculating rates, to avoid overestimation of pedestrian travel injuries.

### Weaknesses

Dustbin coding hindered interpretation of casualty data for all three modes. It was impossible to separate drivers from passengers completely, or on-highway cyclist falls from non-transport falls; nor were all pedestrian falls on-highway identified. Certain sex- and age-specific groups have wide confidence intervals due to small numbers of travellers, especially female fatality rates for walking and cycling, and male fatality rates for cycling, especially older cyclists. This is despite the three year sample period. Although postcode of residence for the numerator data would allow assessment of the area deprivation of those killed or injured, the exposure data did not include socioeconomic data, precluding assessment of socio-economic distribution of injury and fatality rates. This would be important to explore in future studies because of the well known social inequalities in traffic casualties [30], [31].

It is important to recognise that additional bias occurs from not distinguishing between motorway and local driving, with the latter an order of magnitude more dangerous than the former [32]. Casualty rates should ideally compare either driving and cycling on general-purpose roads only, or should compare all driving with, for example, travelling by bicycle for short- and train for long-distance journeys. Given the inclusion of long distance driving in our data, and therefore an underestimation of the risk for driving on local streets, it may be that the crossover for fatality risk between male cyclists and drivers occurs at an older age. Alternatively, cyclists should be compared with low-mileage drivers, who face risks 15–100% greater than the average [33].

The variation in risk was substantial for hospital admissions per billion km, with walking and cycling having rates more than 100 times greater than for driving in some age groups. However, this is not a fair comparison, due to large differences in the mobility achieved using different modes of travel. Differences in injury severity by mode exacerbate the problems of making like-for-like comparisons. Most cyclists ride at moderate speeds for distances <8 km on urban or quieter rural roads [12]. The risks in this paper should not be applied to sportive cycling, mountain biking or BMX riding.

The comparison with published age-specific data from the Netherlands, converted to f/mhu, had a number of limitations, as detailed in the legend to Figure 2. First, De Hartog *et al* compared car users (not drivers) with cyclists [20], thereby diluting the age effect of drivers, as about 25% of car user km are by passengers. Secondly, we were unable to compare sex-specific rates. Cycling in England is dominated by males, raising the overall average risk, whereas cycling distance is split equally between males and females in the Netherlands. Because of the greater risk among males, this increases all-ages average cyclists’ risk in England by one third. Despite these limitations, we found a similar pattern for f/mhu by age and mode of travel in both countries: a marked increase in fatalities in the oldest age groups, particularly for cyclists, and fatality rates for younger car users exceeding those for cyclists for those under 30 y.

Whereas the increased injury and fatality rates among the youngest adults are associated with increased risk of collision, the biggest problem for older people is an increased case-fatality rate [34]. However, although data are sparse for walking or cycling, at least one study has reported increased risk of collisions for older pedestrians and cyclists, which they attributed to a range of age-related factors affecting behaviour [35].

This study did not account for the third parties killed or seriously injured in collisions. This would have had an effect almost exclusively on the driver risks, since pedestrians and cyclists seldom impose risk on others. A previous study has shown that in the UK, such inclusion made all-ages risks per hour similar for cyclists and drivers [24]. In England during the three years of this study, there were nearly 1,000 third party or passenger fatalities in crashes that involved at least one male driver aged 17–20 y (personal communication from Department for Transport to MW, 12/10/12). This means that there were almost two deaths of others for every fatality of a male driver aged 17–20 y. Further research is required to clarify the extent to which young males are a direct cause of fatalities amongst other road users.

### Conclusions

This paper examined the risks of walking, cycling and driving in England using comparable categories of casualty from mortality statistics and hospital records, for age- and sex-specific groups. Cycling is no longer an activity predominantly of youth but it remains very male-dominated, at 80% of distance cycled per year in the UK. The risks per hour of the three modes vary within similar ranges, but show different trends with age. Notably, males aged 17–20 y faced almost five times greater risk per hour as drivers than as cyclists; in contrast, male cyclists 70+ were the most at risk of any group in this study. Females of all ages faced low risks relative to males, except for older drivers.

Comparisons with the Netherlands revealed a similar pattern by age in both countries for both cyclists and car users/drivers. Fatality rates by time travelled in the Netherlands were lower for cyclists than for car users in age groups under 50 y, but for older age groups cycling risk increased sharply, as in England.

Fatality rates by distance travelled were similar for cyclists and pedestrians when collisions and falls on-highway were included, but were generally an order of magnitude higher than for drivers. The important exception was for males under 20 y, in whom the fatality rates as drivers/car users were higher than as cyclists.

Results for driver risks did not include third parties killed or seriously injured in collisions. Not making like-for-like comparisons for numerator data and type of road combine to sustain the myth that cycling is relatively hazardous.

## Supporting Information

Table S1 **ICD-10 coding groups used for transport modal risk study**
(XLS)
